# Organic Molecular Glues to Design Three-Dimensional Cubic Nano-assemblies of Magnetic Nanoparticles

**DOI:** 10.1021/acs.chemmater.4c00770

**Published:** 2024-07-11

**Authors:** Mohammad Suman Chowdhury, Daniel Arenas Esteban, Rabia Amin, Claudia Román-Freijeiro, Enja Laureen Rösch, Markus Etzkorn, Meinhard Schilling, Frank Ludwig, Sara Bals, Verónica Salgueiriño, Aidin Lak

**Affiliations:** † Institute for Electrical Measurement Science and Fundamental Electrical Engineering and Laboratory for Emerging Nanometrology (LENA), Hans-Sommer-Str. 66, Braunschweig 38106, Germany; ‡ EMAT, University of Antwerp, Groenenborgerlaan 171, Antwerp B-2020, Belgium; § CINBIO, Universidade de Vigo, Vigo 36310, Spain; ∥ Institute of Applied Physics, TU Braunschweig, Mendelssohnstraße 2, Braunschweig 38106, Germany; △ Departamento de Física Aplicada, Universidade de Vigo, Vigo 36310, Spain

## Abstract

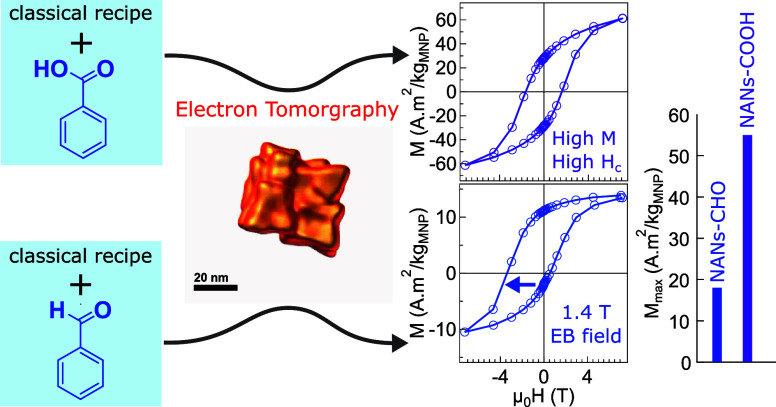

Self-assembled magnetic nanoparticles offer next-generation materials that allow harnessing of their physicochemical properties for many applications. However, how three-dimensional nanoassemblies of magnetic nanoparticles can be synthesized in one-pot synthesis without excessive postsynthesis processes is still a bottleneck. Here, we propose a panel of small organic molecules that glue nanoparticle crystallites during the growth of particles to form large nanoassembled nanoparticles (NANs). We find that both carbonyl and carboxyl functional groups, presenting in benzaldehyde and benzoic acid, respectively, are needed to anchor with metal ions, while aromatic rings are needed to create NANs through π–π stacking. When benzyl alcohol, lacking carbonyl and carboxyl groups, is employed, no NANs are formed. NANs formed by benzoic acid reveal a unique combination of high magnetization and coercivity, whereas NANs formed by benzaldehyde show the largest exchange bias reported in nanoparticles. Surprisingly, our NANs show unconventional colloidal stability due to their unique nanoporous architectures.

## Introduction

Metal oxide nanoparticles are functional materials^1^ where a wide variety of metal ions can be incorporated^2^ to harness the broad physicochemical properties of particles for catalysis,^3^ optoelectronics,^4^ data storage,^5^ diagnosis,^6^ and therapy.^7−9^ It will become exceedingly appealing and open novel functionalities if nanoassembly of these nanoparticles can be achieved in a facile and one-pot synthesis, where properties from building blocks or nanocrystallites and nanoassembled particles are combined in a single entity and the colloidal stability of the nanoassembled particles is not compromised. Using nanoparticles, different classes of colloidal assemblies have been reported.^10^ To make the assembled structures, a range of techniques have been employed including coulomb interaction,^11,12^ shape-controlled,^13^ crystallization,^14^ interparticle linking chemistry,^15^ stabilizer desorption,^16^ and template-assisted techniques.^17^ However, they all require a multistep preparation and, for some, a complex work up to get the final structures, limiting their widespread applications in innovative applied research studies. Some multicore nanoparticles can, however, be prepared in one-pot, but these particles exhibit only semispherical shapes and are not self-assembled.^18−20^ The particle shape can be crucial when a guided orientation,^21^ improved therapeutic effects,^13,22,23^ high MRI contrast,^24^ collective magnetic couplings,^9,25^ and/or a high surface area for more chemical functional groups for bioconjugation is desired.

In this study, using a panel of small organic molecular glues (OMGs) containing carbonyl or carboxyl and phenyl groups, we show how nanoassembled nanoparticles (NANs) with three-dimensional (3D) nanoporous architectures can be designed in a one-pot facile synthesis that requires no excessive work up and postsynthesis processes. The OMGs glue nanobuilding blocks into large cubes when nanobuilding blocks are formed during the thermal decomposition of metal precursors. OMGs lacking carbonyl or carboxyl moieties showed no NANs. In addition, because of the OMG-facilitated NAN formation, the NANs feature nanoporous structures that give them an unconventional colloidal stability despite being 54 nm large.

## Methods

### Chemicals

Cobalt(II) acetylacetonate (≥99.0%), iron(III) acetylacetonate (trace metal based, 99.9%), oleic acid (OA, 90%), dibenzyl ether (DBE, ≥ 98%), 1-octadecene (ODE, 90%), benzaldehyde (≥99%), benzoic acid (≥99.5%), and benzyl alcohol (≥99%) were purchased from Sigma-Aldrich. Methanol, chloroform, isopropanol, and ethanol with the highest purity grade were obtained from Carlroth, Germany. Sodium oleate (>97%) was purchased from TCI, America. All chemicals and solvents were used without further purification.

### Synthesis of Nanoparticles

The synthesis procedures of nanoassembled nanocubes (NANs), classical nanoparticles (NPs-Classic), and crystallite nanoparticles (NPs-Crystallite) were adapted from our previous work with some modifications.^2^ Briefly, 1 mmol (353.0 mg) of iron(III) acetylacetonate, 1 mmol (257.15 mg) of cobalt(II) acetylacetonate, 1 mmol (305.0 mg) of sodium oleate, DBE and ODE 5 mL each, and 4 mmol (1.26 mL) of oleic acid were added into a three-neck 50 mL round-bottom flask. For the synthesis of NPs-Crystallite, the amount of cobalt(II) acetylacetonate was reduced to 0.25 mmol, and all other parameters were unchanged. We highly recommend our readers to read the synthesis section of our previous work for the detailed working protocol.^2^

For degassing with a high vacuum oil pump, the mixture was heated to 90 °C at a heating ramp rate of 3 °C/min and kept at that temperature for 65 min. Afterward, the flask was brought under a N_2_ environment. Immediately after, to synthesize the NANs, 1 mmol of organic molecular glue (OMG) was added to the reaction mixture. Next, without changing the heating ramp rate, the mixture was heated to 130 °C and kept at this temperature for 5 min. Afterward, a heating ramp rate of 2 °C/min was applied to reach the nucleation and growth temperature of 290 °C and the reaction was continued at that temperature for an additional 30 min. Note, the heating temperature, heating ramp, and soak time for different steps were set on a PID temperature controller prior to starting the reaction, and no further modifications were made during the reaction.

The crude synthesis mixture was brought to ambient conditions and the work up was performed according to our previous work.^2^ Finally, the obtained nanoparticles were stored at room temperature in chloroform.

### Synthesis of 35 nm Nanoparticles

To synthesize 35 nm large nanoparticles, 1 mmol (353.0 mg) of iron(III) acetylacetonate, 1 mmol (257.15 mg) of cobalt(II) acetylacetonate, 10 mL of DBE, and 4.5 mmol (1.43 mL) of oleic acid were added into a three-neck 50 mL round-bottom flask. Afterward, for degassing with a high vacuum oil pump, the mixture was heated to 90 °C at a heating ramp rate of 4 °C/min and kept at that temperature for 60 min. Afterward, the flask was brought under a N_2_ environment. Next, without changing the heating ramp rate, the mixture was heated up to 130 °C and kept at this temperature for 5 min. Afterward, a heating ramp rate of 20 °C/min was applied to reach the nucleation and growth temperature of 290 °C and the reaction was continued at that temperature for an additional 30 min. The crude synthesis mixture was brought to ambient conditions and the work up was performed according to our previous work.^2^ Finally, the obtained nanoparticles were stored at room temperature in chloroform.

### Characterization Methods

#### Transmission Electron Microscopy (TEM)

Transmission electron microscopy studies were carried out using a JEOL TEM microscope operating at 100 kV. The samples were prepared by drop casting 5 μL of particle suspension in chloroform on a grid (Formvar-carbon coated copper grids with the mesh size of 300) and letting it to completely dry under a fume hood. The TEM images were analyzed by using ImageJ software.

#### High-Angle Annular Dark-Field Scanning Transmission Electron Microscopy (HAADF-STEM) and Energy Dispersive X-ray Spectroscopy (EDX)

High angle annular dark-field scanning transmission electron microscopy (HAADF-STEM) and energy dispersive X-ray spectroscopy (EDX) have been performed on an aberration-corrected cubed FEI Titan microscope equipped with a Super X EDS detector operating at 300 kV. EDS analysis were performed by acquiring at least 100 frames of 1024 × 1024 pixels resolution by using a dwell time of 5 μs at a higher current of 100 pA to ensure enough counts.

Electron tomography tilt series were acquired using a Fischione 2020 tomography holder. A high-resolution HAADF-STEM tilt series was acquired over a tilt range between ±70° with a tilt increment of 2°. The obtained series were aligned using cross-correlation and 3D reconstructions were obtained using the expectation maximization (EM) algorithm, as implemented in Astra Toolbox.^26,27^

#### Inductively Coupled Plasma Optical Emission Spectroscopy (ICP-OES)

ICP-OES analyses were performed on a Varian (715ES) instrument. The sample preparation is as follows: 25 μL of particle suspension in chloroform was added into a 10 mL volumetric flask, and then 1 mL of aqua regia (HCl: HNO_3_ @ 3:1) was pipetted into the flask. Next, the sample was incubated for 1 h at ≈60 °C to facilitate the digestion process and then left overnight inside a fume hood. Next day, Milli-Q water was added to the flask up to the 10 mL grading level. The calibration curves were prepared prior to the measurement of every sample set.

#### Attenuated Total Reflectance-Fourier Transform Infrared (ATR-FTIR) Spectroscopy

ATR-FTIR studies were carried out by using a Bruker VERTEX 70 spectrometer. The samples were prepared by drop casting 5 μL of particle suspension in chloroform on the diamond crystal of the ATR-FTIR and letting it completely dry. After that, 12 scans were performed to obtain the data.

#### Raman Spectroscopy

Raman spectra were obtained with a Renishaw inVia Reflex confocal system. Experiments were conducted at room temperature using a 532 nm excitation wavelength (Nd:YAG/Nd:YVO_4_ diode laser). The spectra were obtained over an acquisition time of 120 s and 1% laser power.

#### Magnetic Property Measurement System (MPMS)

The magnetization hysteresis loops were measured by using MPMS (Quantum Design). The samples were prepared by pipetting a 15 uL of particle suspension in chloroform at a particle concentration of 3 mg/mL into a designated measurement capsule and letting it thoroughly dry. The hysteresis loops were recorded at 298 K in magnetic fields between −7 and 7 T. The ZFC-FC measurements were recorded between 5 and 380 K in 5 mT magnetic fields. The field-cooled (FC) hysteresis loops were recorded after cooling the samples in 5 T saturating magnetic fields to the desired temperature. The applied magnetic fields were corrected for a remanence field in the chamber and the magnets by measuring the palladium standard sample. The coercive fields were corrected accordingly. The magnetization values originally recorded in the units of emu (electromagnetic units) were normalized to the amount of iron obtained from the ICP-OES analysis.

#### Alternating Current Susceptometry (ACS)

ACS was performed using a home-built setup operating at the ac magnetic field amplitude of 95 μT and a frequency ranging from 200 Hz to 1 MHz. The measurements were performed on 150 μL of particle suspensions in chloroform at 298 K at different particle volume fractions.

## Results and Discussion

**Figure 1:**
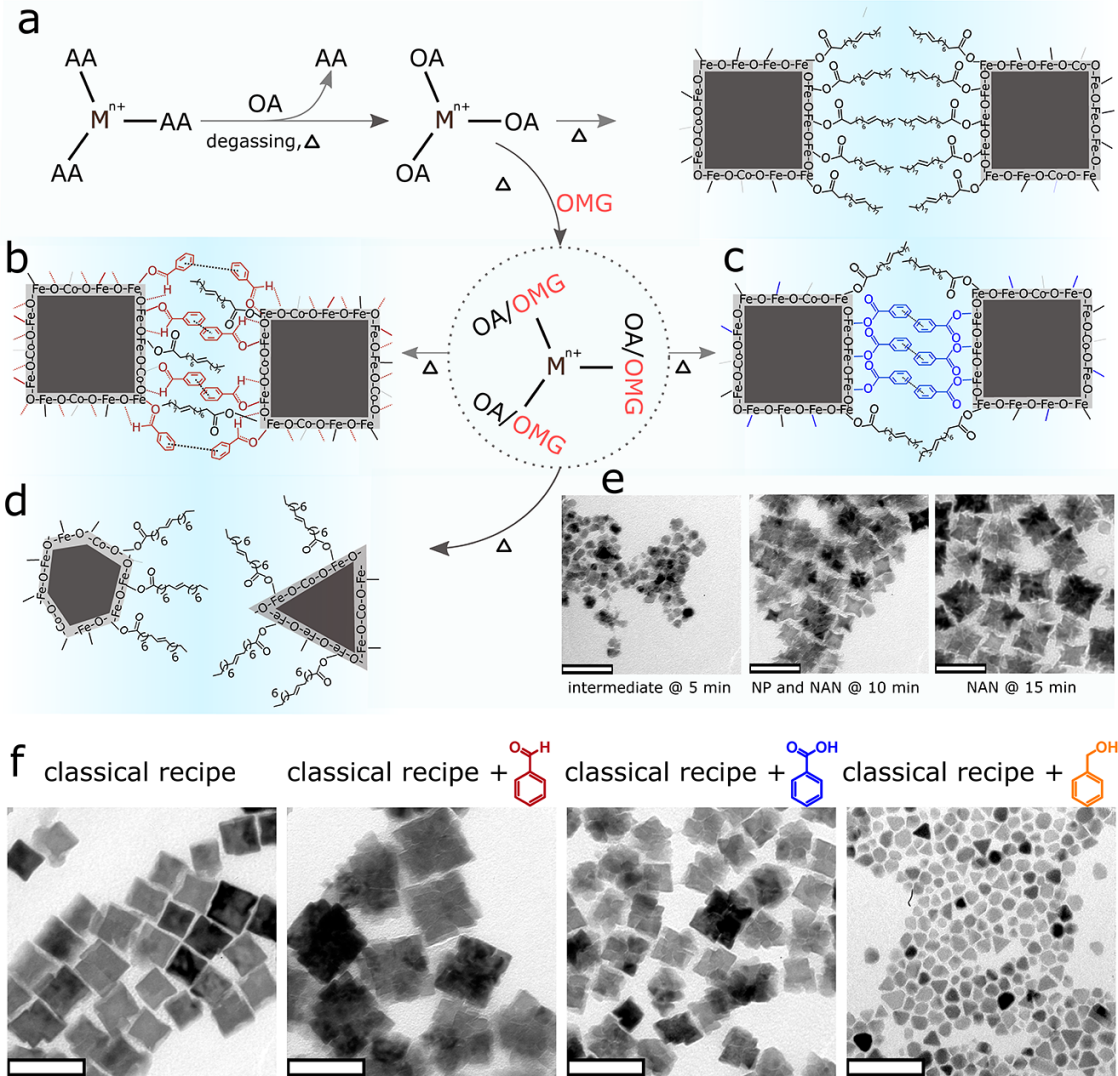
Influence of organic molecular glue (OMG) on formation of nanoassembled nanoparticles. (a) Scheme showing acetylacetonate (AA) ligands and metal ion with different valencies (M^n+^) that loses AA when mixed with oleic acid (OA) and degassed at 90 °C under high vacuum conditions. With further heating to 290 °C, single cubic nanoparticles are formed over 30 min (top right). (b) When benzaldehyde is added as an OMG after degassing, its aldehyde group coordinates with the metal ions and oxygen atom through hard Lewis-acid base and hydrogen bonding, respectively. Additionally, its phenyl group facilitates π–π stacking between nanobuilding blocks. (c) Similarly, when benzoic acid is added instead of benzaldehyde, its carboxyl group coordinates with the metal ions in monodentate, and/or bidentate, and bridging fashion and the phenyl ring leads to π–π stacking between nanobuilding blocks. (d) When benzyl alcohol is added, it does not glue nanoparticle crystallites; instead, it acts as a solvent which leads to tetrahedron and rombohedron nanoparticles. (e) TEM-based growth mechanism demonstrating major steps prior to nanoassembled nanoparticles (NANs) formation. During the soak time at 290 °C, small intermediate nucleates are formed after ∼5 min; after 10 min, both single nanobuilding blocks and NANs can be seen; and after 15 min, all the nanobuiliding blocks are glued to form large single NANs. (f) TEM micrographs of single nanocubes synthesized using classical recipe (NPs-Classic), NANs synthesized using aldehyde (NANs-CHO), NANs synthesized using benzoic acid (NANs-COOH), and NPs synthesized using benzyl alcohol (NPs–OH), respectively, from left to right. The classical recipe includes oleic acid, sodium oleate, octadecene, and dibenzyl ether. All of the scale bars correspond to 60 nm.

**Figure 2:**
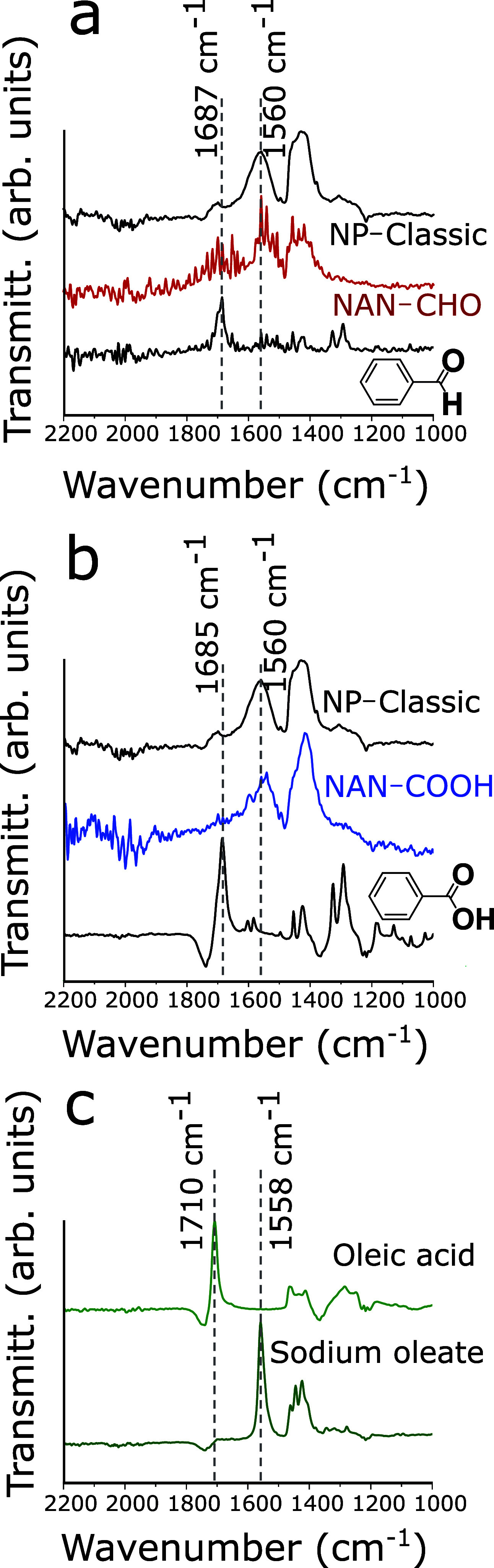
ATR**-**FTIR transmittance spectra of nanoparticles and NANs. (a) The top panel shows the spectrum of NPs-Classic, the middle panel shows the spectrum of NANs-CHO, and the bottom panel shows the spectrum of benzaldedhyde. (b) The top panel shows the spectrum of NPs-Classic, the middle panel shows the spectrum of NANs-COOH, and the bottom panel shows the spectrum of benzoic acid. (c) The top panel shows the spectrum of oleic acid, and the bottom panel shows the spectrum of sodium oleate. The ATR-FTIR spectra of NPs-Classic is presented twice in (a) and (b) for a better comparison.

Thermal decomposition-based synthesis of magnetic nanoparticles (MNPs) requires extensive degassing to remove (i) precursor coordinating ligands such as acetylacetonate, (ii) volatile compounds that coexist in reagents used, and (iii) organics that are generated due to decomposition of solvent. A compromised degassing process dramatically influences the nucleation, growth, shape, and size of magnetic particles.^28,29^ Knowing that, we designed our synthesis in which we add small organic molecules that might appear in synthesis due to solvent decomposition.^30^ The role such small molecules play in controlling the nucleation and growth kinetics of MNPs has been very puzzling for quite some time. Using iron acetylacetonate as precursor and oleic acid as capping ligand, typically oleic acid replaces acetylacetonate during degassing and initial heating. When heating at high temperature is pursued, single core nanoparticles are formed (Figure 1a,f). Our initial experiments to unravel the role of small organic molecules led to serendipity, which unveiled mysterious effects of small coordinating molecules on the synthesis of MNPs. One of those molecules is benzaldehyde, which carries a carbonyl moiety and a hydrogen atom linked to it. We observed several intriguing features in our synthesis. Transmission electron microscopy (TEM) studies show that upon addition of benzaldehyde to the synthesis, the resulting particles display a nanoassembled nanoparticle (NAN) morphology instead of a classical single-core nanoparticle morphology (Figure 1b,f). To gain more insights, we experimented with other small molecules, all having an aromatic ring but differing in functional groups. By adding benzoic acid, analogous to benzaldehyde but bearing a −OH group linked to its carbonyl moiety, we obtained comparable NANs (Figure 1c,f). However, when benzyl alcohol—lacking a carbonyl moiety but bearing a −OH group—is added, no NANs are formed (Figure 1d,f). Each of these three small molecules carries an aromatic ring, and thus they are comparable except their functional groups. Therefore, having seen their gluing properties, we call them organic molecular glues (OMG). To elucidate the growth mechanism of these OMG-induced NANs, we collected aliquots at different growth time points. Surprisingly, it unfolded some unique features. First, small star-shaped building blocks are formed. Second, the building blocks start to join with one another and form more and more cubic-shaped NANs with time. Third, when all the nanobuilding blocks are consumed, the nanobuilding blocks within the NANs reorganize themselves effectively and form more ordered structures. Interestingly, all of these occur within a 15 min time span (Figure 1e). The formation mechanism of such NANs is unique, and remarkably, the NANs exhibit an overall cubic morphology (Figure 1f). It has been demonstrated that intermolecular π–π stacking, despite being relatively weaker than hydrogen or covalent bonds, plays a crucial role in supramolecular assembly.^31^ In addition, iron ions being hard Lewis acid interact strongly with hard Lewis bases such as the oxygen atom.^32^ Therefore, we hypothesize that both benzaldehyde and benzoic acid due to their carbonyl and carboxyl functional groups interact with the metal ions through the hard Lewis acid–base interaction in the first stage of particle growth. Additionally, due to their smaller sizes compared to oleic acid, they reduce the steric hindrance at the surface of the nanobuilding blocks. Consequently, within the first 15 min, the nanobuilding blocks are formed and get glued together through intermolecular π–π interactions between the aromatic rings (Figure 1b,c,e). However, with benzoic acid, we obtained smaller NANs compared with benzaldehyde-doped synthesis (Figure 1b,c). On the other hand, benzyl alcohol acts like a good ion dispersing solvent that promotes the nucleation and growth of particles and ultimately leads to particles with different morphologies including tetrahedron and rombohedron and high polydispersity (Figure 1d,f). This is often seen when particles are synthesized by the polyol-based method.^20^ Therefore, it becomes more rational that both aldehyde and carboxyl functionalities are inevitable to preserve the morphology of the nanobuilding blocks and the aromatic ring is inevitable to glue the nanobuilding blocks together through π–π interaction to produce the NANs. Additionally, we investigated the reproducibility of our synthetic procedure by synthesizing another batch of NANs doped with benzaldehyde. Indeed, it confirmed that our synthesis procedure is repeatable (Figure S1). It is worth noting that washing the NANs with highly polar solvent such as methanol or isopropanol or with a mixture of both, or even harsh sonication, does not disassemble the NANs into single nanobuilding blocks, further validating the presence of π–π interactions. Additionally, we performed attenuated total reflectance-Fourier transform infrared (ATR-FTIR) spectroscopy measurements that partially revealed the presence of the OMGs. We find that when benzaldehyde is used in the synthesis, the NANs show a stretching vibration of the carbonyl group at 1687 cm^–1^ that overlaps with the pristine carbonyl signal of benzaldehyde (Figure 2a). This suggests that benzaldehyde is present in the NANs which is not seen in the IR spectrum of the NPs-Classic, showing no band at 1687 cm^–1^ (Figure 2a). Structurally, unlike the carboxylic acid group, the aldehyde group in benzaldehyde can exclusively interact with metal ions through hard Lewis acid–base interaction as there is no possibility of having an ionic form of aldehyde moiety. However, in the case of NANs synthesized with benzoic acid, we could not see any peak at 1685 cm^–1^, which is seen with the pristine benzoic acid molecule (Figure 2b). This can be attributed to the fact that the COOH group is converted into its ionic COO^–^ moiety and demonstrates a significant redshift to 1560 cm^–1^ after interacting with the metal ions through both hard Lewis acid–base and ionic interactions (Figure 2b). Such a shift can be conveniently understood if the carbonyl bands of oleic acid and sodium oleate are compared. Sodium oleate, which is the ionic form of oleic acid, displays the carbonyl band at 1558 cm^–1^, whereas the charge neutral form of the oleic acid generates the carbonyl band at 1710 cm^–1^ instead of 1558 cm^–1^ (Figure 2c). Therefore, the presence of benzoic acid in the NANs remained ambiguous. However, comparing the morphologies of the NANs-COOH and NPs-Classic, it is plausible to assume that benzoic acid should be present between the nanobuilding blocks.

**Figure 3:**
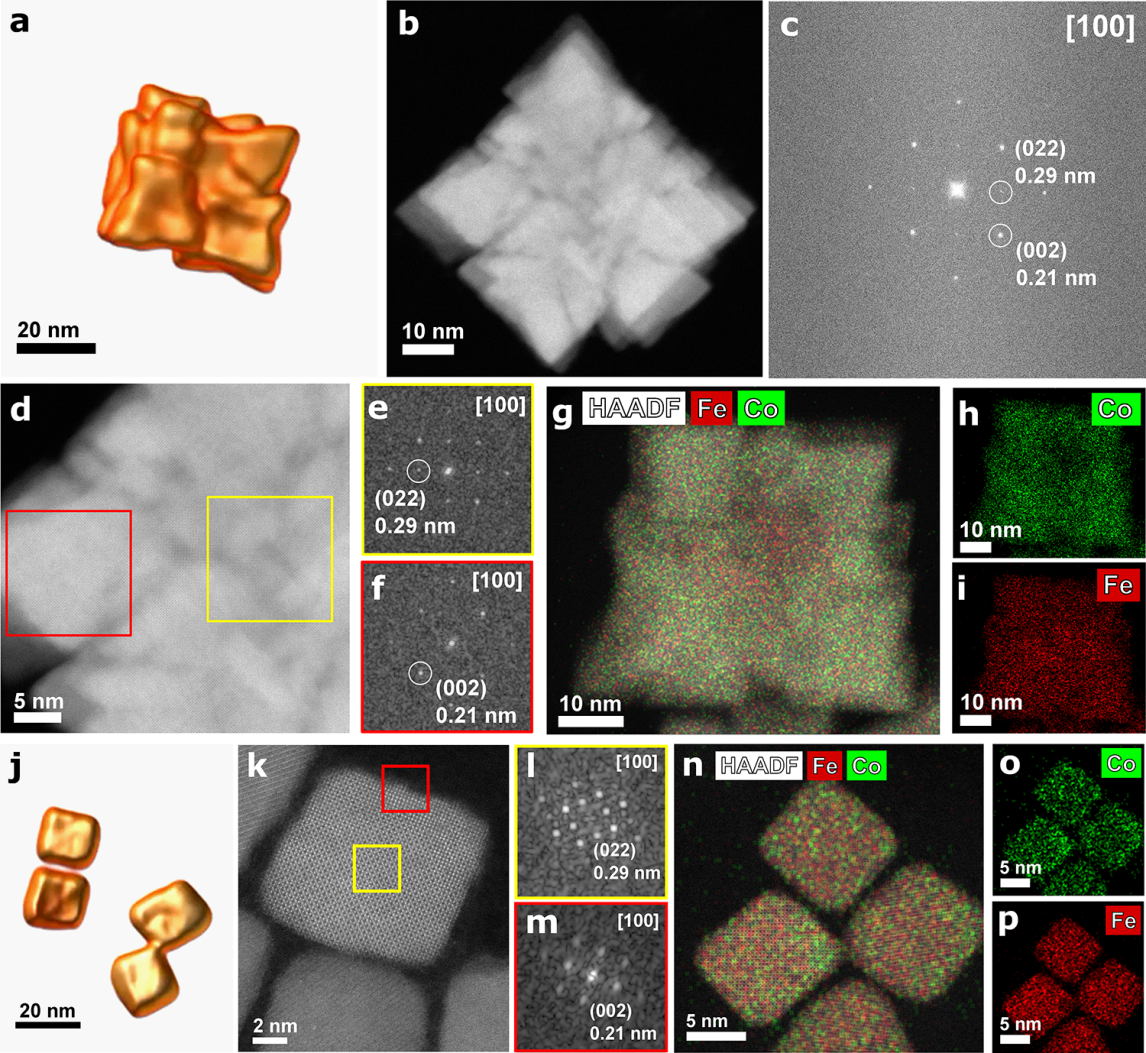
(a) 3D visualization of a single NANs-CHO reconstructed using electron tomography. (b) HAADF-STEM image of a single NANs-CHO clearly showing the organization of the nanobuilding blocks into a large cubic NAN. (c) Fast Fourier transform (FFT) of the HAADF image shown in panel (b), revealing the arrangement of the building blocks with precision along a unified crystal orientation within a single NAN. (d) High-magnification HAADF-STEM image from the particle shown in panel (b). (e, f) The FFT analyses were performed on two regions highlighted by yellow and red squares, showing different crystal structures. The FFT pattern shown in panel (e) is characteristic of a spinel phase with the cubic crystal structure. The FFT pattern shown in panel (f) can be attributed to a rock-salt crystal structure. (g–i) EDX elemental maps of a NANs-CHO showing that the particle core is more populated with Fe and the particle external borders have more Co. (j) 3D visualization of the nanobuilding blocks (NPs-Crystallite) reconstructed using electron tomography showing their cubic morphology. (k) High-resolution HAADF-STEM image of the nanobuilding blocks and (l, m) their respective FFT patterns from the areas highlighted by yellow and red squares in panel (k). (n–p) EDX elemental maps of the nanobuilding blocks, where the cobalt-rich regions can be observed at the outer borders of the particles.

We then sought to unravel how the building blocks are glued together to form the NANs. To shed light on their organization within the NANs, we performed electron tomography in high-angle annular dark-field scanning transmission electron microscopy (HAADF-STEM) mode, high-resolution HAADF-STEM, and energy-dispersive X-ray (EDX) analysis on NANs-CHO and the building blocks (NPs-Crystallite). 3D visualization of the NANs-CHO obtained by electron tomography revealed that the NANs-CHO are composed of ∼8–10 building blocks (cf. video in the supplementary file), with an overall cubic morphology and generates interspacings that vary from 0.7 to 1.0 nm (Figure 3a, b). This falls within the range of 0.846 to 1.13 nm, which is the calculated length of sandwich and parallel-displaced dimers of two aromatic rings of the glue molecules.^33,34^ The range, however, does not match the length of oleic acid that is ∼2.08 nm,^35^ suggesting the qualitative presence of the glue molecules at the interspaces. The fast Fourier transform (FFT) obtained from the image in Figure 3b reveals a distinct and well-defined crystalline pattern (Figure 3c), indicating that the constituent building blocks are arranged with precision along a unified crystal orientation within a single NAN by sharing their basal planes. Higher resolution HAADF-STEM analyses, however, revealed distinct structural differences within a single NAN (Figure 3b). Looking more closely at the FFT analyses, two different crystal lattices can be indexed to the center and the external borders of a single NAN (highlighted by red and yellow squares) (Figure 3d). We found that the center of the NANs has a spinel crystal structure that can be assigned to cubic *Fd*3*m̅* space group 227 (Figure 3e). Interestingly, the external border has a rock-salt crystal structure with cubic *Fm*3*m̅* space group number 225 (Figure 3f). Energy-dispersive X-ray (EDX) studies confirmed the FFT analyses, indicating that the NANs-CHO particles are rich in Co and Fe at the external border and at the center of the NAN, respectively (Figure 3g–i). A plausible composition of the cobalt-rich phase with the rock-salt crystal structure can be CoO. By reducing the feed of Co precursor and not adding the OMGs, we synthesized 9 nm NPs-Crystallite with cubic morphology that resemble the nanobuilding blocks of NANs and enable comparing their physical properties (Figure 3j, cf. Video S1). The FFT analyses of HAADF-STEM micrograph align with the cubic spinel phase of Co_*x*_Fe_2–*x*_O_4_ (*x* = 0.5, based on ICP, Table 1), while some external boundaries occasionally display traces of rock-salt crystal structures in a minor fraction (Figure 3k–m). The EDX analyses demonstrate that metal distribution is generally uniform within the particles. Nevertheless, it is notable that certain outer regions of the particles show Co enrichment, in agreement with the minor rock-salt structures found at the external borders (Figure 3n,o).

**Table 1:** Feed and Final Compositions of Co and Fe in Nanoparticles (NPs) and Nanoassemblies, Their Physical Size ± Standard Deviation (SD) (Size Histograms Are Shown in Figure S2), Maximum Magnetization *M*_max_ ± SD at 298 K, Coercive μ_0_*H*_c_ ± SD at 5 K and Exchange Bias μ_0_*H*_EB_ ± SD at 20 K field values, and blocking temperature *T*_B_ ± SD as derived from ICP-OES, TEM, Magnetization Hysteresis Loops in Zero-Field-Cooled (ZFC) and Field-Cooled (FC) Modes, and Temperature-Dependent ZFC-FC Magnetization Measurements and Analyses^a^

Nanocubes (NCs)	Fe/Co (feed ratio in mmol)	NCs composition (based on ICP)	NCs size (nm)	*M*_max_ (A·m^**2**^/kg_MNP_) @ 298 K	*μ*_*0*_*H*_c_ (mT) @5 K	*μ*_*0*_*H*_EB_ (mT)@20 K	*T*_B_ (K)
NPs-Classic	1.0/1.0	Co_**1.28**_Fe_**1.72**_O_**4**_	25.0 ± 7.5	22.2 ± 1.5	1103 ± 60	914.7 ± 107.1	331.7 ± 0.7
NPs-Crystallite	1.0/0.25	Co_**0.50**_Fe_**2.5**_O_**4**_	9.0 ± 2.0	63.9 ± 0.2	1748 ± 40	0	226.7 ± 0.2
NANs-CHO	1.0/1.0	Co_**1.33**_Fe_**1.67**_O_**4**_	54.0 ± 10.5	18.7 ± 0.5	958 ± 12	1241.8 ± 161.9	238.9 ± 1.0
NANs-COOH	1.0/1.0	Co_**1.03**_Fe_**1.97**_O_**4**_	28.0 ± 9.3	54.5 ± 0.7	1599 ± 23	127.6 ± 4.0	N.D.
NPs–OH	1.0/1.0	Co_**0.79**_Fe_**2.21**_O_**4**_	10.6 ± 0.2	69.6 ± 1.8	1557 ± 24	0	247.9 ± 0.7

a Measurement uncertainities are determined from three independent measurements. N.D. stands for not determined.

**Figure 4:**
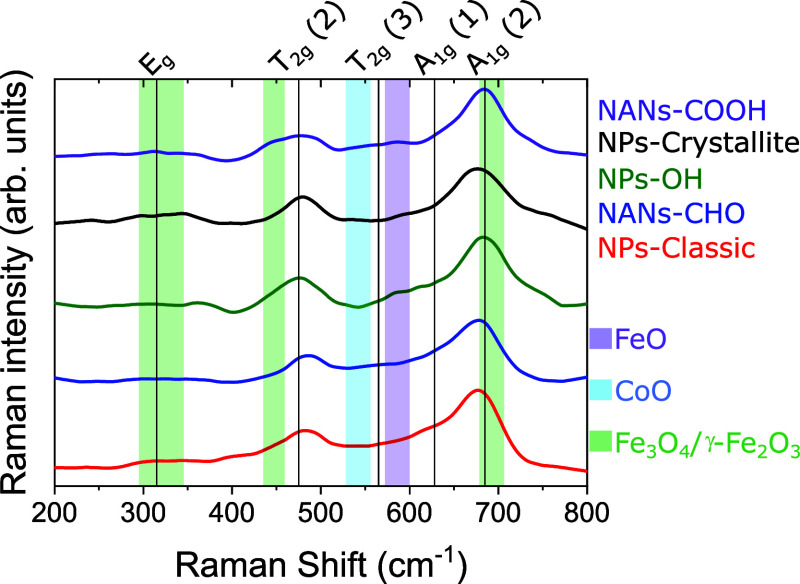
Raman spectra of all of the samples obtained using a 532 nm excitation wavelength. Five of the six vibrational modes of the cobalt ferrite spinel structure are indicated by black vertical lines. Secondary phases are indicated with shadowed areas in light blue (CoO), violet (FeO), and light green (Fe_3_O_4_/γ-Fe_2_O_3_).

To determine different crystalline phases that seem to coexist in the samples, we performed Raman spectroscopy using a 532 nm excitation wavelength (Figure 4). According to group theory, the spinel ferrite crystalline structure (space group *Fd*3̅*m*) exhibits the following optical phonon modes:
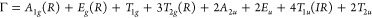
out of which five are Raman active (indicated with (*R*)).

In the case of the cobalt ferrite, six main features centered around 180, 315, 475, 565, 628, and 685 cm^–1^ are expected, corresponding to the vibrational modes T_2g_(1), E_g_, T_2g_(2), T_2g_(3), A_1g_(1), and A_1g_(2), respectively (indicated five of them by black vertical lines in Figure 4). The A_1g_ phonon mode is usually found to split into two different modes, that are centered at 628 and 685 cm^–1^. The splitting of the A_1g_ mode in the spectrum of CoFe_2_O_4_ NPs indicates the presence of Co^2+^ and Fe^3+^ cations being incorporated into the spinel structure. Previous studies have addressed the origin of the relative intensity between the two A_1g_ components,^36,37^ associating the higher-frequency A_1g_ phonon mode to the trivalent iron cations in tetrahedral A interstitial sites, and the lower-frequency A_1g_ component was directly correlated to the presence of Co^2+^ cations in the same interstices. This fact is also related to the high intensity registered for the T_2g_(2) vibrational mode, which has been reported to originate solely from the Co^2+^ cations in the octahedral B interstitial sites. This band is typically overshadowed in the case of Fe_3_O_4_.^38^ Accordingly, the Raman spectra of all the five samples reveal the normal spinel crystalline structure expected for Co_*x*_Fe_3–*x*_O_4_ as the dominant, with a varying amount of Co^2+^ in the tetrahedral A sites. In this regard, looking at the relative reduced intensity of the T_2g_(2) mode, using the A_1g_(2) vibration mode as a reference, we can infer that there is a very low percentage of Co^2+^ in the octahedral B sites in all the samples. Specifically, the NPs-Classic and NANs-CHO samples seem to have the lowest amount of Co^2+^ in the spinel structure, suggesting that a fraction of Co^2+^ must be in other forms. The Co^2+^ in the A sites seems to be the highest in the NPs–OH and NPs-Crystallite samples. The spectrum of NANs-COOH differs from that of NANs-CHO, indicating the presence of other secondary phases in the former sample.

Furthermore, given the information provided by HAADF-STEM, we next analyzed the spectra by paying attention to the possibility of detecting FeO or CoO. The rock-salt centrosymmetric crystal structure of these oxides is a weak Raman scatterer, yet two-phonon Raman scattering is permitted. Additionally, though forbidden, the first-order phonon scattering can occur because of the presence of cobalt vacancies or structural defects, recurrently presenting in nanostructures.^39^ The presence of a rather weak vibrational band at 540 cm^–1^ (shadowed in light blue) can be attributed to this one-phonon longitudinal optical mode of the CoO.^40^ Provided that the possible structural defects in the rock-salt phase at the external boundaries, seen in the HAADF-STEM studies, contribute minimally to this band, there is a major fraction of CoO phase in the NPs-Classic and NANs-CHO and less significant in the NANs-COOH. Likewise, the presence of a weak vibrational mode at 585 cm^–1^ (shadowed in light violet) can indicate the presence of FeO in the NPs-Crystallite, NANs-COOH, and NPs–OH. The Raman spectra have other overshadowed areas (in light green) at 300–350, 450 and 665–700 cm^–1^, matching different vibration modes of iron oxides (magnetite/maghemite) with the spinel crystalline structure.^41^ These bands are most visible for NANs-COOH and NPs–OH and least recognizable for the NANs-CHO.

**Figure 5:**
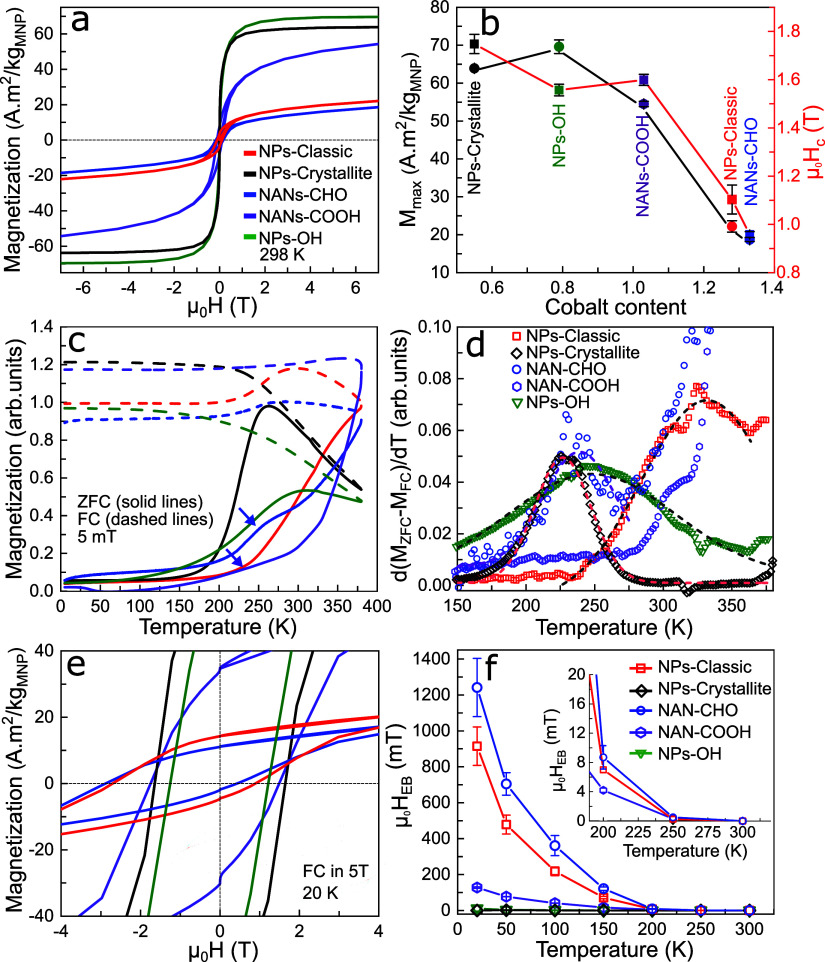
(a) Magnetization hysteresis loops measured at 298 K and plotted as an average of three independent measurements. (b) Maximum magnetization (*M*_max_) and coercive field (*H*_c_) values versus the cobalt content *x*. The values are mean ± SD and were determined from three independent measurements. (c) Zero-field-cooled (ZFC) and field-cooled (FC) magnetization curves measured at 5 mT as a function of temperature varying from 5 to 380 K. (d) d(*M*_ZFC_ – *M*_FC_)/d*T* vs *T* plotted over a limited range for the sake of clarity. The dashed gray and colored lines are the best Gaussian fits to a limited range of the data. For the sake of clarity, the fits are not shown for all the samples. (e) Magnetization hysteresis loops recorded at 20 K after cooling the sample in 5 T cooling fields (FC) to 20 K. The curves are the average of three measurements. (f) Exchange bias field (*H*_EB_), given by *H*_EB_ = −(H_+_ + H_–_)/2, extracted from the FC hysteresis loops shown in panel (e) vs *T*. The range between 200 and 300 K is zoomed in and shown as inset. The EB values are mean ± SD determined from three measurements. The same color coding is applied to all of the panels. All of the measurements were carried out in a vibrating sample magnetometer (VSM) mode on samples dried from particle suspensions in chloroform at a particle concentration of 3 g/L. Measurement uncertainities of magnetic parameters are given in Table 1.

To understand how the coexistence of different phases and unique ordering of the nanobuilding blocks within the NANs are reflected in magnetic properties, we performed various magnetic measurements. We observed a complex correlation betweenthe cobalt content *x*, the nature of added OMG, the particle size, and magnetic properties. Looking at the magnetization hysteresis loops recorded at 298 K (Figure 5a) on dried particles, we found that maximum magnetization (*M*_max_) depends nonmonotonously on the cobalt content *x* (Figure 5b). Tuning the *x* from 0.50 in the NPs-Crystallite to 0.79 in the NPs–OH by increasing the cobalt feed level and adding benzyl alcohol increases *M*_max_ from (63.9 ± 0.24) to (69.6 ± 1.8) A·m^2^/kg, approaching the bulk values at 300 K.^42^ An increased magnetization by doping more Co^2+^ into iron oxide structure suggests that the NPs–OH is mainly Co-doped γ-Fe_2_O_3_, as also reported by Fantechi et al.^43^ The presence of a small fraction of the Fe_3_O_4_/γ-Fe_2_O_3_ phase in this sample, as deduced from the Raman band at 450 cm^–1^, may also contribute to the increase of the magnetization. The *M*_max_ drops to (54.5 ± 0.7) A·m^2^/kg by increasing *x* to 1.03 in the NANs-COOH, corresponding to a total 15% drop in magnetization within the doping from *x* = 0.50 to 1.03; whereas a 30% drop in single-core NPs within the same range was reported by Sathya et al.^44^ This may be partially due to the presence of Fe_3_O_4_/γ-Fe_2_O_3_ phase in the NANs-COOH. Considering spin-only magnetic moments of 3 μ_B_ for Co^2+^ and 4 μ_B_ for Fe^2+^, the drop in magnetization by further replacement of Fe^2+^ with Co^2+^ is expected.^43^ An ordered assembly of the nanobuilding blocks into nanoporous cubic assemblies in the NANs-COOH may lead to magnetic dipolar interactions between the building blocks, which in turn increase the net magnetic moment of the NANs.^13,45^*M*_max_ drops for both NPs-Classic and NANs-CHO samples, in which *x* increases to values above *>*1. The Raman and magnetic results show that the addition of benzaldehyde as an OMG forms antiferromagnetic (AFM) CoO as the major Co-phase, reducing the magnetization in these two samples. The presence of Co-rich phase at the outer layers of the NANs-CHO has also been observed in the HAADF-STEM analyses (Figure 3g–i). Seeing no saturation magnetization at 7 T for both NANs resonates with the presence of AFM FeO and CoO in NANs-COOH and CoO in the NANs-CHO.

The coercive field (*H*_c_) at 5 K, plotted as a function of *x*, decreases with the particle size, with the NANs-COOH representing an exception.^44^ Single-domain to multi-domain particle transition in Co_*x*_Fe_3–*x*_O_4_ NPs occurs at around a particle size of 20 nm.^46^ In the multi-domain regime, *H*_c_ drops with the particle size. The NANs-COOH, despite being 28 nm, have μ_0_*H*_c_ = (1599 ± 23) mT, which is much larger than μ_0_*H*_c_ = (1103 ± 60 mT) of 25 nm NPs-Classic. This demonstrates that the NANs does not fall into the traditional definition of multi-domain NPs. We propose that the enhanced *H*_c_ is the result of the nanobuilding blocks being well-ordered within the NANs, coinciding with the findings of Peddis et al. in centrosymmetric nanoassemblies of CoFe_2_O_4_ crystallites.^47^ The NANs-CHO particles show a similar trend. Their *H*_c_ is comparable with that of NPs-Classic (Table 1), despite being nearly twice the size of the single-core NPs-Classic. However, the *H*_c_ decreases significantly since the Co comes mainly as the AFM CoO phase in NANs-CHO, which has a much lower coercivity than CoFe_2_O_4_. Within the NANs, our data show that *H*_c_ is mainly governed by ordering and phase composition of the nanobuilding blocks rather than their overall physical size. Putting all together, using benzoic acid as an OMG, a unique combination of high *M*_max_ and extremely high *H*_c_ can be achieved in a single nanoassembly. Of note, the volume *V* and the magnetic moment *m* = *M* × *V* of a cubic NANs-COOH with the side length *L* are (6/π) times of a spherical NP with the diameter *D*, provided that *L* = *D*. Despite their *M*_max_ being ∼60% of defect-free Fe_3_O_4_ NPs, i.e., *M*_s_ = 94 A·m^2^/kg,^48^ thanks to their cubic morphology, the NANs-COOH have a larger *m* than defect-free Fe_3_O_4_ NPs of the same size.

We next performed zero-field-cooled (ZFC) and field-cooled (FC) temperature dependent magnetic measurements to shed light on magnetic relaxation processes of the NANs (Figure 5c). For data analysis, we looked at d(*M*_ZFC_ – *M*_FC_)/d*T* vs *T* plot (Figure 5d), as proposed by Bruvera et al. as the “good” method for determination of the blocking temperature *T*_B_.^49,50^ Remarkably, the NANs-CHO show a distinct magnetic transition shoulder at ∼240 K (Figure 5c, shown by the blue arrow). We mainly attribute this transition to the *T*_B_ of the nanobuilding blocks rather than to the Néel transition from AFM to paramagnetic in CoO phase which occurs at *T*_N_ = 291 K.^51^ The relaxation processes of the nanobuilding blocks strongly suggests that the building blocks are, to some extent, decoupled from each other within the NANs.^52^ The *T*_B_ of the nanobuilding blocks matches the *T*_B_ of NPs-Crystallite, indicating that these two have a comparable magnetic volume and magnetic anisotropy. We, however, cannot completely rule out a contribution from the Néel transition in CoO phase to the increase of the ZFC branch up to ∼240 K. *M*_ZFC_ branch of the NANs-CHO steadily rises and shows no reversible magnetization from 240 to 380 K, suggesting that the NANs-CHO, as a whole entity, should have a *T*_B_ at *T* > 380 K (Figure 5d). Similarly, two-step relaxation processes, however less pronounced, can be discerned for NANs-COOH (purple arrows in Figure 5c). Our data strongly suggest the coexistence of decoupled and coupled building blocks in the NANs. The ZFC curve of the 25 nm NPs-Classic shows a one-step steady rise with a mean *T*_B_ of 331.7 K (gray dashed line, Figure 5d), typical of single-core MNPs. In contrast to the NANs-CHO, the NPs-Classic do not show any magnetic transition at 240 K, despite having a comparable fraction of the AFM CoO phase (Figure 4), further indicating a negligible role of the Néel transition in CoO in this magnetic transition. These data show that not only their crystal structures but also their magnetic domains have no nanoassembled organization.

We made interesting and supportive observations in the magnetization hysteresis loops recorded at 20 K after cooling the samples in 5 T saturating magnetic fields, the so-called FC hysteresis loops (Figure 5e). The shift in the FC loops is the fingerprint of an exchange coupling process called exchange bias (EB).^53−55^ The exchange bias has been reported in different classes of biphasic magnetic NPs.^56,57^ While the NPs-Crystallite and NPs–OH samples revealed no exchange bias, we observed an exceptionally large μ_0_*H*_EB_ = (1241.8 ± 161.9) mT at 20 K in the NANs-CHO, which is still detectable at *T* > 200 K (Figure 5f, inset, Table 1). Considering that the *T*_N_ of FeO and CoO phases are 198 and 291 K, respectively,^51^ our FC measurements suggest that the NANs-CHO should have a major fraction of AFM CoO phase. These results support the Raman findings, wherein no detectable fraction of the FeO phase was seen in this sample. The presence of a mixed AFM Fe_*x*_Co_1–*x*_O cannot, however, be totally ruled out based on our data. Such a phase has been shown to form in the cobalt-doped iron oxide NPs.^58^ We hypothesize that because of nanoporous structures, high interfacial volume, and CoO AFM phase the NANs-CHO show such large exchange bias. Zakutna et al. have recently reported a μ_0_*H*_EB_ = 1100 mT for cobalt-doped iron oxide NPs,^59^ which is to the best of our knowledge the highest value thus far reported. In contrast, the single-core NPs-Classic despite having crystalline phases similar to the NANs-CHO possess a significantly lower μ_0_*H*_EB_ = (914.7 ± 107.1) mT at 20 K, supporting our conclusion on the positive effect of the nanoporosity on the exchange bias (Table 1).

**Figure 6:**
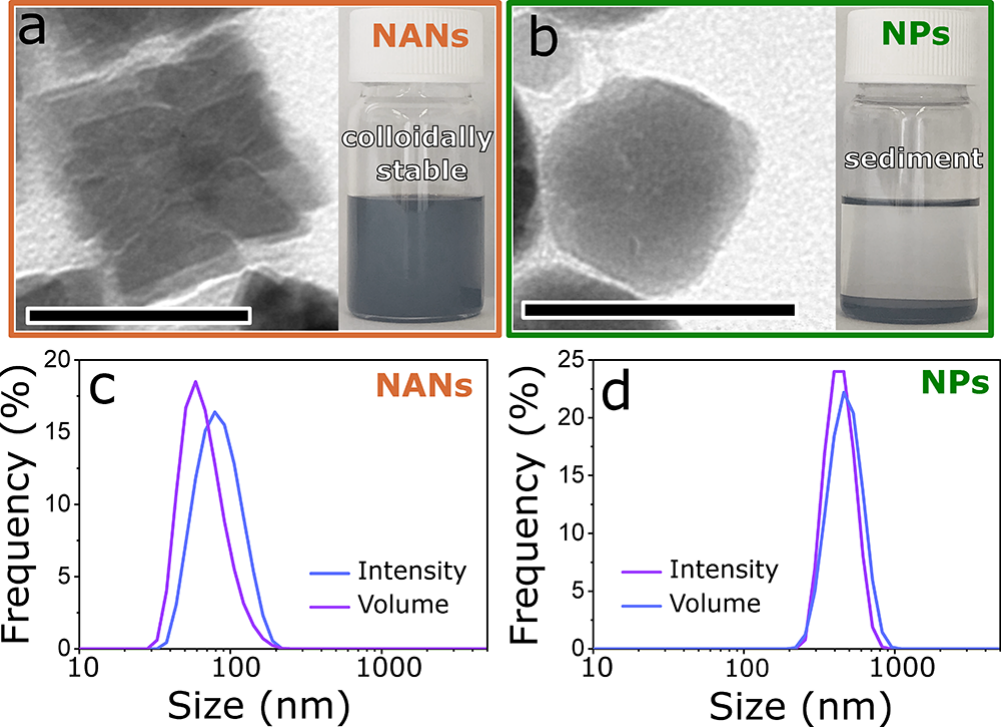
Characterization of the colloidal stability of NANs and NPs in chloroform. Digital photographs of the particles in glass vials were taken 5 min after the full particle dispersion in solvent was achieved. (a) TEM image of a single NAN (on the left) and the digital photograph of colloidally stable NANs (on the right). (b) TEM image of a single single-core NP (on the left) and the digital photograph of sedimented single-core NPs (on the right). Scale bars: 50 nm. Panels (c) and (d) show the volume- and intensity-weighted dynamic light scattering (DLS) size distributions of NANs and NPs, respectively. The size histograms are the averages of three measurments.

Use of MNPs as reporters for magnetic biosensing and as heat mediators for magnetic hyperthermia requires four key features, but not limited to (i) high saturation magnetization, (ii) large magnetic volume, (iii) excellent colloidal stability, and (iv) controlled surface functionalization. However, MNPs larger than 25–30 nm tend to rapidly aggregate due to strong magnetic dipole–dipole interactions.^2,29^ Consequently, the colloidal stability and surface functionalization of MNPs are highly compromised, which makes the MNPs unsuitable for downstream biological applications. Remarkably, our NANs, despite being 54 nm large, preserve their colloidal stability in contrast to the single-core NPs of 35 nm (Figure 6a,b). Although both NANs and single-core NPs are coated with oleic acid surfactant molecules that are hydrophobic in nature, the NANs show no sedimentation in chloroform over 6 months, whereas the single-core NPs show sedimentation in chloroform within 5 min. Our work demonstrates that the role of OMGs in creating nanoporous architectures in the NANs is key, which further gives them an exceptional colloidal stability and thus prevents their aggregation. To quantify their colloidal stability in chloroform and tendency toward aggregation, we analyzed the NANs and the single-core NPs by dynamic light scattering (DLS) (Figure 6c,d). We find that the volume- and intensity-weighted means of the NANs are (68.9 ± 24.6) (mean ± σ) and (86.8 ± 29.1) nm, respectively. In contrast, the volume- and intensity-weighted means of the single-core NPs are (479.9 ± 119.3) and (441.4 ± 97.1) nm, respectively. The DLS data clearly suggest that NANs have excellent colloidal stability as they do not aggregate/cluster in chloroform; whereas, the single-core NPs show poor colloidal stability as they aggregate/cluster in chlorofrom. To examine if the particle concentration influences magnetization dynamics of the NANs, we performed alternating current susceptometry (ACS) measurements on particle suspensions in chloroform at 3 (0.06 v%) and 23 g/L (0.46 v%). The ACS data, shown in Figure S3, show a characteristic single magnetic relaxation peak in imaginary part χ″ at ∼2.2 kHz. The peak position is at ω × τ_B_ = 1, with ω being the angular frequency and τ_B_ the Brownian relaxation time constant. The relaxation peak frequency is highly sensitive to particle colloidal stability and clustering.^60^ The peak shifts toward lower frequency or slower Brownian relaxation when particles are clustered. Looking at Figure S3, we find that the relaxation peak remains unchanged although the particle volume fraction increases from 0.06 v% to 0.46 v%. This indicates that the NANs do not magnetically interact even at a high-volume fraction.

## Conclusions

Here, we provide mechanistic insights into the design of ordered 3D nanoassemblies that capitalize on a panel of small organic molecules. We show that both benzaldehyde and benzoic acid, despite being small molecules, can effectively create NANs; while analogous benzyl alcohol-based synthesis creates tetrahedron and rombohedron nanoparticles but no NANs. The key benefits of the NANs are, but not limited to (i) no postsynthesis functionalization is needed for their assembly, (ii) no complex work up is needed, and (iii) no aggregation occurs despite being 54 nm large. Their exceptional colloidal stability in solution investigated by DLS can mainly be attributed to their nanoporous architectures. The NANs reveal unconventional magnetic properties, nonexistent in the discrete building blocks. Benzoic acid doped NANs show a combination of high magnetization and magnetic moment and a very high coercive field. In contrast, benzaldehyde doped NANs show the highest exchange bias ever reported in nanoparticle-based magnetic systems by promoting the formation of the CoO phase as a major secondary phase and offering a large AFM-F(i)M interfacial volume due to their nanoporosity. We believe that our approach to synthesize 3D assemblies in situ can be expanded to particles with other metal oxides to combine different properties in a single particle system. Additionally, our work demonstrates the overlooked role of small organic molecules in designing next-generation inorganic nanomaterials with nanoporous architectures and thus sets the foundation to explore other organic molecules. Furthermore, we believe that as the NANs exhibit high colloidal stability in their pristine state in chloroform, they can be transferred to aqueous medium as single particles as well and, for example, can be applied for magnetic immunoassays where single particles in aqueous medium are needed to capitalize on the Brownian relaxation properties of the single particles pre- and postanalyte binding.

## Supporting Information Available

Chemicals, nanoparticle synthesis procedures, characterization methods of particles, Figure S1 showing TEM image of reproduced NANs, Figure S2 showing size histograms, and Figure S3 showing ACS spectra at different particle volume fractions (PDF)
3D electron tomography video of the NANs and nanobuilding blocks (AVI)

## Supplementary Material

cm4c00770_si_001.pdf

cm4c00770_si_002.avi
