# Supplementary material for: Organic Molecular Glues to Design Three-Dimensional Cubic Nano-assemblies of Magnetic Nanoparticles
Source: Chem Mater. 2024 Jul 11;36(14):6865–76. doi: 10.1021/acs.chemmater.4c00770 (PMC11270742; doi:10.1021/acs.chemmater.4c00770)
Supplement: Supplementary file 1 — cm4c00770_si_001.pdf [file cm4c00770_si_001.pdf]

Supplementary Information for

Organic Molecular Glues to Design Three-Dimensional Cubic Nano-assemblies of Magnetic Nanoparticles

Mohammad Suman Chowdhury[a], Daniel Arenas Esteban[b], Rabia Amin[a], Claudia Román-Freijeiro[c], Enja Laureen Rösch[a], Markus Etzkorn[d], Meinhard Schilling[a], Frank Ludwig[a], Sara Bals[b], Verónica Salgueiriño[c], Aidin Lak[*a]

a. Institute for Electrical Measurement Science and Fundamental Electrical Engineering and Laboratory for Emerging Nanometrology (LENA), Hans-Sommer-Str. 66, Braunschweig, 38106, Germany

b. EMAT, University of Antwerp, Groenenborgerlaan 171, B-2020 Antwerp, Belgium

c. CINBIO, Universidade de Vigo, 36310 Vigo, Spain

d. Institute of Applied Physics, TU Braunschweig, Mendelssohnstraße 2, 38106, Braunschweig, Germany

Email: a.lak@tu-braunschweig.de

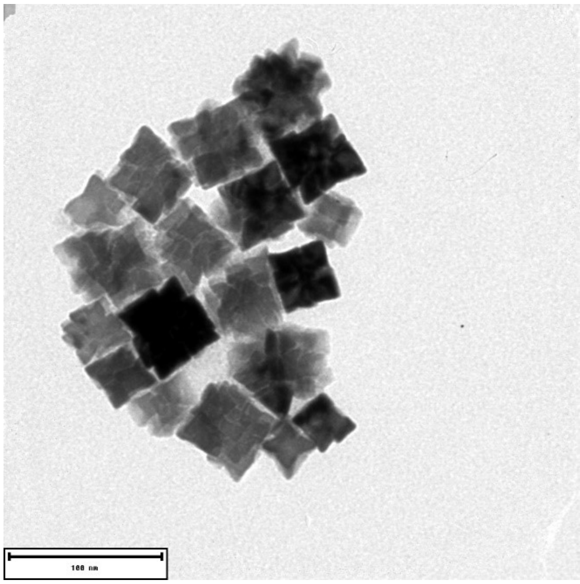

Supplementary Figure 1. TEM micrograph of NANs showing the reproducibility of NANs-CHO. Scale bar corresponds to 100 nm.

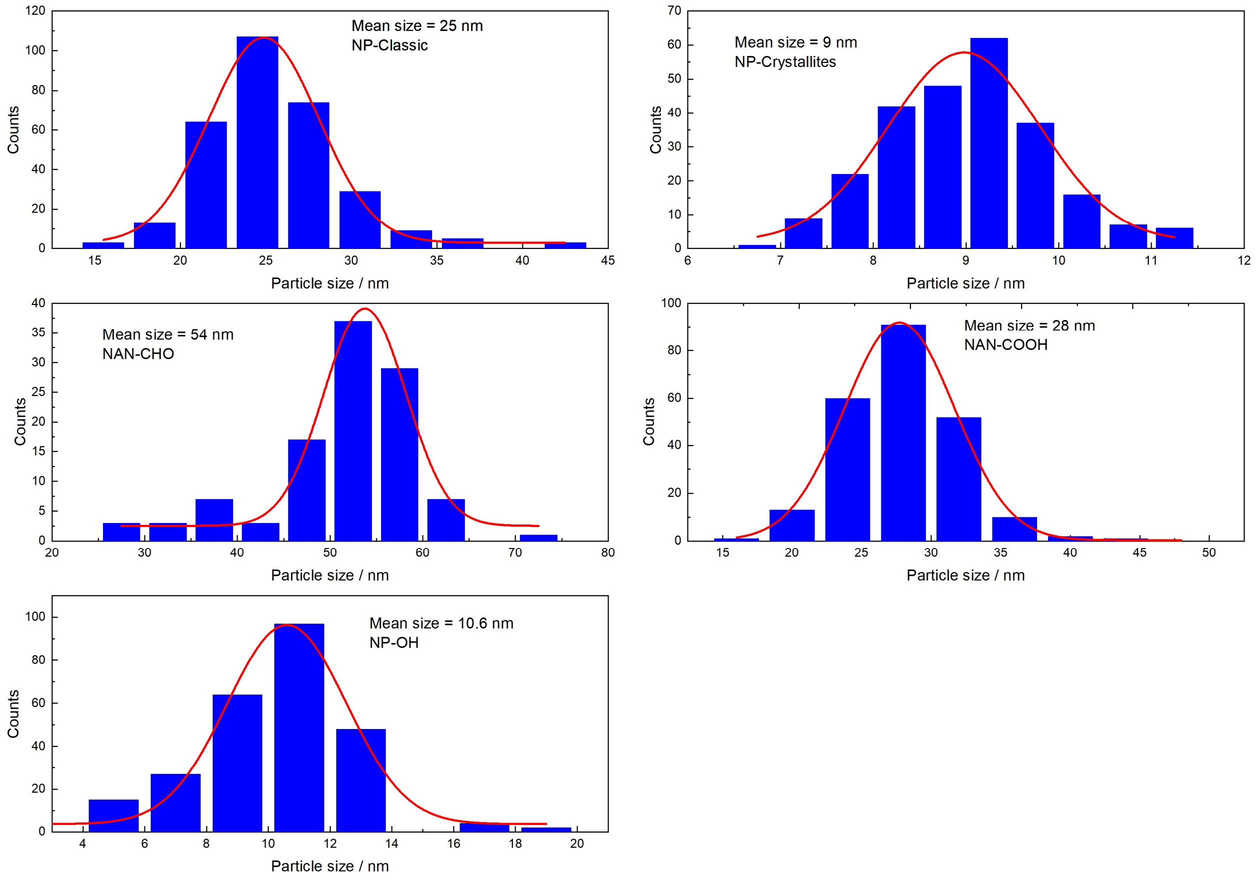

Supplementary Figure 2. Histograms of particle size distribution. N = 100-300 nanoparticles for size distribution analysis. The bin sizes are varied from 0.5 to 5 for a proper Gaussian fit.

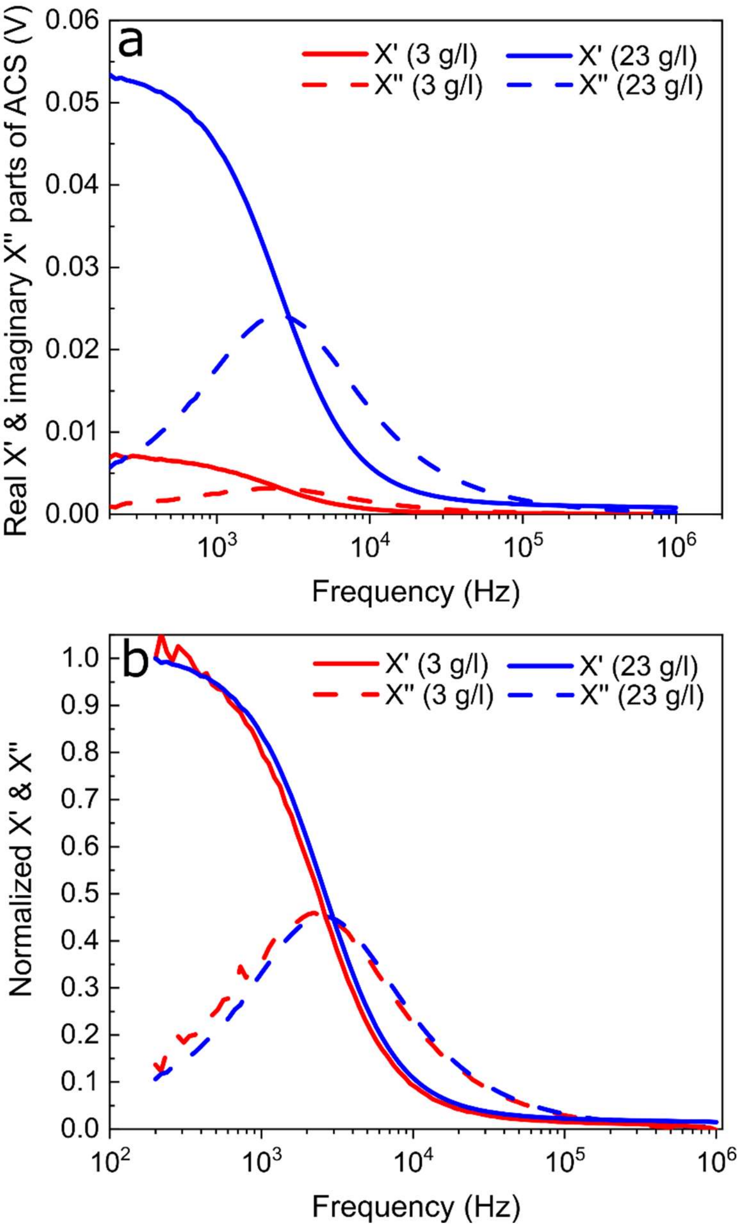

Supplementary Figure 3. Alternating current susceptometry (ACS) spectra of NANs-CHO particle suspensions in chloroform at 3 g/l (0.06 v%) and 23 g/l (0.46 v%). The measurements were performed on 150 µl of particle suspensions at 298 K and 95 µT ac magnetic fields. The relaxation peaks seen in χ´´ show the Brownian relaxation of these MNPs. With low and high particle volume fractions, there is no shift between the relaxation peak frequencies, indicating no significant magnetic dipolar interactions in the NANs even at high particle volume fraction.
